# Spiritual care to persons with dementia in nursing homes; a qualitative study of nurses and care workers experiences

**DOI:** 10.1186/s12912-015-0122-6

**Published:** 2015-12-28

**Authors:** Liv Skomakerstuen Ødbehr, Kari Kvigne, Solveig Hauge, Lars Johan Danbolt

**Affiliations:** Department of Nursing, Faculty of Public Health, Hedmark University College, PO Box 400, N-2418 Elverum, Norway; Institute of Health and Society, Department of Nursing Science, University of Oslo, PO Box 1130, Blindern, 0318 Oslo, Norway; Nesna, University College, Institute of Nurse Education, 8700 Nesna, Norway; Institute of Health Sciences, Faculty of Health and Social Studies, Telemark University College, 3901 Porsgrunn, Norway; Centre of Caring Research – Southern Norway, Institute of Health Sciences, Faculty of Health and Social Studies, Telemark University College, 3901 Porsgrunn, Norway; Centre for Psychology and Religion, Innlandet Hospital Trust, PO Box 68, 2312 Ottestad, Norway; Oslo School of Theology, PO Box 1153, Blindern, 0318 Oslo, Norway

**Keywords:** Dementia, Focus groups, Nursing homes, Nursing practice, Spiritual care

## Abstract

**Background:**

Spiritual care for people with dementia who are in nursing homes is one aspect of the holistic care provided by nurses. A number of studies have explored the concepts of spirituality and religiosity, but fewer studies describe how nurses provide spiritual care in practice. The Purpose of the study was thus to investigate how nurses and care workers can provide spiritual care for people with dementia who live in nursing homes.

**Methods:**

This is a qualitative study with an exploratory design using a phenomenological–hermeneutic approach. Interviews were conducted in eight focus groups with 31 nurses and care workers in 4 Norwegian nursing homes.

**Results:**

The nurses were unsure about whether they actually provided spiritual care. Through discussions in the focus groups, a new understanding and insight was developed. The spiritual care that the nurses provided included: (1) integrating spiritual care into general care, described as ‘physical touch’ and ‘responsiveness and intuition’; (2) spiritual care in terms of togetherness, described as 'being present’ and 'sensitivity in communication’; and (3) spiritual care as providing meaningful activities for everyday life, described as 'facilitating activities’ and 'meeting the residents’ religious needs’.

**Conclusions:**

This study demonstrates the need for nurses and care workers to discuss and reflect on how to understand and describe spiritual care for people with dementia in practice. There is a need to develop and expand the knowledge about how to teach carers to recognize resident’s spiritual needs and expressions of spirituality and to establish a comprehensive view of spiritual care for people with dementia in nursing homes.

## Background

Life expectancy in the Norwegian population has increased continually over recent decades [1]. Increased life expectancy is also a global trend arising from improvements in health care that have allowed people to live longer, healthier lives [2]. However, this change has resulted in additional challenges among the oldest old people because the prevalence of dementia increases with age [2]. Dementia affects the brain, making people vulnerable and unable to provide detailed information about themselves [3]. The characteristics of dementia are memory impairment and impaired communication and orientation, which lead to difficulties functioning in society [4]. People with dementia have an extensive need for care, whether physical, mental, social or spiritual, throughout the course of the disease [4]. This fact may lead to growing health care needs in older populations in the future [2]. Many people in Norway who have dementia spend their last days in nursing homes [5].

Spiritual concerns are particularly evident among older people (aged 65 years+) [6, 7]. Spiritual care is part of the holistic care that nurses provide [8–10], but for people with dementia, providing spiritual care is particularly challenging as a result of their cognitive decline, which leaves many affected people unable to talk about their spirituality [11, 12]. A relevant concern is how research defines spiritual care and, furthermore, how nurses can meet the spiritual needs of people with dementia. Sawatzky and Pesut [13] define spiritual care as

“An intuitive, interpersonal, altruistic, and integrative expression that rests on the nurses’ awareness of the transcendent dimension yet reflects the patients’ reality.” (p. 30)

Sawatzky and Pesut [13] highlight the significance of altruistic care, which implies that nurses show attitudes such as cheerfulness, compassion and kindness, love, joy and peace in their encounters with residents. Studies in dementia care emphasize interpersonal perspectives, in line with Sawatsky and Pesut’s [13] definition, by focusing on the good relationships between nurses and residents; in doing so, these studies indicate that confirming the resident’s personhood is part of spiritual care [14–17]. The definition of Sawatzky and Pesut [13] indicates that dementia care is asymmetrical, and the nurses are the strong party in the interaction. Nurses’ attitudes are thus important making demands on the nurses in showing respect and taking in to account the residents vulnerability. In addition, Sawatzky and Pesut [13] claim that spiritual care is an integrative expression that rests on nurses’ awareness of residents’ transcendent dimensions. The word ‘transcendent’ means ‘something out of the ordinary in a particular object or experience’ (p. 39) [18]. Transcendent experiences are often associated with experiences of faith in God/a deity, meaningfulness and something exceeding understanding [18–20]. Studies in dementia nursing emphasize helping residents listen to music, participate in memory work, receive sensory stimulation, or express faith and beliefs as a way of facilitating such moments [21–24]. Furthermore, in research, transcendent moments describe people’s experiences of connectedness in relationships, e.g., with themselves, other people and the environment [25–28].

It is difficult for nurses to immerse themselves in a resident’s reality when the resident has dementia and cognitive decline. Nurses attempt to understand residents’ spirituality, which implies that they must interpret residents’ spiritual expressions. Another challenge that nurses face when providing spiritual care is related to the abstract nature of descriptions of spirituality, which blur the understanding of spiritual care [29]. This lack of clarity has led nurses to find spiritual care demanding and to report a lack of understanding and knowledge about how to provide spiritual care in practice [30, 31].

This review of current research on spiritual care in dementia care shows that several questions need to be addressed regarding how to transfer and apply knowledge about spiritual care to people with dementia in nursing homes.

### Aim

The aim of this study was to investigate how nurses and care workers provide spiritual care for people with dementia in nursing homes.

## Methods

### Study design

We conducted a qualitative study with an exploratory design. To gain access to nurses’ ‘lived experiences’ regarding the provision of spiritual care in dementia settings, a phenomenological–hermeneutic approach was employed [32]. Phenomenological methodology provides the opportunity to expand the understanding of an investigated phenomenon. Phenomenology enables researchers to examine nurses’ lived experiences of the phenomenon ‘providing spiritual care’ in many different ways. In working with the interview text, the researchers’ understanding of the nurses’ lived experiences varied between partial understanding and comprehensive understanding, in line with the hermeneutic circle [33]. Ricœur and Thompson [34] argue that this understanding is related to proximity to and distance from the text when the purpose is to understand its meaning. Comprehension is thus both a fusion of horizons and a conflict between understanding and explanation and between parts of text and the whole text, similar to the hermeneutic approach [33]. In this way, the phenomenological and hermeneutic methodologies complement each other.

Focus groups were used because we wanted to investigate spiritual care in pre-existing groups in which some of the nurses (registered nurses [RNs]) and care workers (licensed practical nurses [LPNs]) already knew each other and had worked together previously. Such ‘natural clusters’ at work provide part of the ‘scripting’ for nursing practice in specific contexts [35]. Another main reason for using focus groups was that the theme was considered difficult to talk about in nursing care, and thus easier to discuss together with others [35].

### Setting

Four nursing homes participated in the study. Information about the study was provided orally and in writing to the leaders of each department, who in turn asked two department members, either nurses or care workers, to voluntarily participate in the study [36]. The inclusion criteria for the participants were twofold: (i) a permanent employee who (ii) had been working at least 1 year in a nursing home and in dementia care. We have suggested that clinical experience affects nurses’ understanding of their 'lived experience’ and their understanding of their patients [37]. Norwegian nursing homes are characterized by small units adapted for people with dementia, all with single rooms [38]. Current research reveals that 80 % of residents in nursing homes have dementia, although not all of them are diagnosed [39].

### Data collection

A total of eight focus group interviews (one first interview and one follow-up focus group interview at each nursing home) were conducted by the same facilitator (first author) and moderator (second author). There were 26 participants in the first focus group interview; 15 of these participants participated in the follow-up focus group interview. We had hoped to include the same participants in both focus group interviews to ensure stability across the groups [40], but because of shift changes, there were five new participants in the follow-up focus group interviews. The number of participants who attended each focus- group interview was between four and eight. Table 1 gives an overview of the participants in focus group interview 1 and those in the follow-up focus group interview.

**Table 1:** Participants in interview 1 and the follow- up interview

Nursing home	Interview 1	Follow-up interview	New participants in the follow-up interview
A: *n*	6	4	2
B: *n*	8	6	0
C: *n*	6	6	3
D: *n*	6	4	0
Total: *n*	26	20	5

Diversity in the focus groups can contribute to variations in the participants’ experiences and give additional nuance to the data; we experienced this in our focus groups [41]. A total of 31 participants (16 nurses and 15 care workers) participated in the investigation; of these, only one was male. Four of the participants were younger than 30 years, and 16 were older than 50 years. Four of the participants had worked fewer than 5 years in dementia units, and 17 had worked more than 10 years. Table 2 provides an overview of the participants’ ages and work experience.

**Table 2:** Participants’ age and working experience

Age (years)	Number	Working experience (years)	Number
<30	4	<5	4
30–50	11	5–10	10
>50	16	>10	17

Each focus group interview lasted 1½–2 h (90–120 min), and was conducted in the meeting room of the nursing home. The workmethod for the focus group session was explained by the facilitator at the start of the session. Written information was given in the form of a thematic guide that contained three questions: ‘What is your understanding of spiritual care?’, ‘How do patients express spiritual needs?’ and ‘What knowledge is important when providing spiritual care?’ The facilitator asked the questions, gave short summaries during the interview and ensured that the conversation did not stop. The moderator played an observer role, conducted unstructured observations, took field notes and asked supplementary questions. The follow-up focus group interview built on the first focus group interview and further explored certain themes. Hummelvoll and Severinson [42] state that re-interviewing participants in focus groups implies that certain topics have developed and that the discussion has deepened during the focus group discussions. Furthermore, re-interviewing allowed the participants to reflect on spiritual care between the first and second focus group interview, which allowed the participants to explore the topic more intensely and deepen their understanding of the theme being investigated.

All of the focus group interviews were audio-recorded and transcribed. The first focus group interview was transcribed before the follow-up focus group interview to ensure continuity and the pursuit of individual topics. The facilitator and moderator discussed the focus group interview afterwards and made notes that were used in the follow-up focus group interview. A summary was written after each focus group interview on the same day and was read at the start of the second focus group interview.

### Ethical approval

Consistent with Norwegian legislation, the collection of data about professional health care workers’ job experiences must be ethically assessed by the Data Protection Official for Research at the Norwegian Social Science Data Services. Permission to conduct the study was granted in April 2011. Participation was voluntary, and written informed consent was obtained from the participants before the study began. The participants’ anonymity was preserved, and all participants could withdraw from the study at any time. The study was based on the World Medical Association’s [43] Declaration of Helsinki statement of ethical principles for medical research involving humans, focusing on safeguarding the integrity of, doing no harm to, and providing dignity and respect for the participants.

### Data analysis

We applied Lindseth and Nordberg’s [32] phenomenological–hermeneutic analysis, which is inspired by the hermeneutics of Gadamer et al. [33] and Ricœur and Thompson [34]. We focused on meaning as experienced by nurses as they investigated what the text stated and their understanding of the text. The analysis focused on the meanings that the nurses and care workers had developed from their experiences with patients, as disclosed during these focus group interviews. The analysis followed three steps [32]:

### Step 1 Naïve reading

The text was read in its entirety to obtain a sense of the whole. During the reading, we wrote down sentences and key words that illustrated the meaning, as we understood it. This naïve understanding guided the structural analysis.

### Step 2 Structural analysis

The entire text was divided into meaningful units. These units were read and reflected on based on the background and our naïve understanding. We then condensed the meaningful units and reflected on their similarities and differences. We sorted similar condensed meaningful units to form subthemes, which were assembled into themes. The themes were reflected on in relation to our naïve understanding. When discrepancies arose, the entire analysis was repeated. Field notes that were written in free-form during the interviews were read retrospectively during the analysis. The notes described the observations, assumptions and experiences of the co-moderator during the interviews and thus provided validation for important points during the data analysis. The themes and subthemes of the structural analysis are presented in the results section and are provided in the vernacular with verbatim quotes for a more accurate reflection of the informants’ experience [32].

### Step 3 Comprehensive understanding

The themes and subthemes were summarized and reflected on in relation to the research question and the context of the study. The text was read then again in its entirety, bearing the naïve understanding and the validated themes in mind. The process of interpreting the text in its entirety and arriving at a comprehensive understanding is what Lindseth and Nordberg [32] refer to as the ‘non-methodical’ pole of understanding (p. 151). This comprehensive understanding is presented at the beginning of the discussion. During the analysis, we were not concerned with comparing the occupational groups; rather, the dialogue within the groups provided the data, regardless of the professional clusters. Consequently, in the results and discussion, we used the term ‘nurses’ generically, including care workers in that designation.

### Rigor and methodological considerations

Credibility and consistency were ensured by asking the same researchers to conduct all interviews and to transcribe, analyse and reflect on the data in a manner that was consistent with our exploration of the method [40]. Furthermore, all four researchers read the transcripts and participated in the analysis. Some researchers consider the phenomenological approach to be incompatible with a focus group method because it is difficult to validate the data with individual informants [44]. However, we have found that focus groups are well suited for examining the lived experience of spiritual care among peers, particularly in nursing contexts, probably because we sought an understanding of the collective, professional life world that the participants shared [42, 45]. The use of the phenomenological methodology meant that we focused on the participants’ lived experience in practice, rather than on abstract considerations of spiritual care. Both the researchers and participants influenced the conversation through their interpretations of each other’s reflections on and representations of spiritual care. The participants experienced an expanded understanding of spiritual care individually and a deeper common understanding collectively. The joint reflection in the focus groups made by nurses from several different contexts created the data, which contribute to strengthen the transferability to other settings. In accordance with the work of Streubert and Carpenter [36] we clarified our own pre-understanding by partially setting aside our own thoughts, ideas and biases to be open to new perspectives.

## Results

The nurses’ provision of spiritual care is outlined in three main themes: (i) integrating spiritual care into general care; (ii) spiritual care in terms of togetherness; and (iii) spiritual care as providing meaningful activities for everyday life.

### Integrating spiritual care into general care

The nurses described spiritual care as an integral part of general care that involved all aspects of the residents’ lives, including helping them eat, dress, bathe, etc. This integration of spiritual care was described as ‘physical touch’ and ‘responsiveness and intuition’.

### Physical touch

The nurses stated that physical touch was an aspect of spiritual care; examples of this type of care included sitting at the resident’s bedside, holding hands, giving a hug and meeting the resident’s eyes while carefully stroking his or her cheek. Physical touch included a friendly smile while washing the resident or rubbing the resident gently with ointments and creams. Physical touch increased the residents’ awareness of themselves, in the sense of ‘here I am – I am me, and here is my body’, as expressed by one of the participants. The manner in which the touch was provided mattered. One nurse expressed it in this way:

D2: ‘There are many residents who appreciate getting a hug, holding hands or being caressed for on their cheek. Some appreciate a good night hug, too.’ (p. 18)

The nurses understood that physical touch created security for the residents, and they believed that the residents experienced touch as calming. Physical touch was a way of confirming the resident as a person and strengthening the resident’s sense of personhood.

### Responsiveness and intuition

The nurses associated spiritual care with a form of intuition. They realized that intuition gave them greater sensitivity to the residents’ needs. The following conversations explored what the nurses meant by intuition:

H2: ‘I think it is to listen and to be responsive and watch the residents’ body language and simply listen.’

H1: ‘If you can sit there and observe, then you use intuition and instincts – it happens automatically, intuition and gut instinct, especially if you do not talk too much.’ (p. 11)

The nurses saw that their awareness of the residents’ behaviour affected how they understood the patients’ spirituality because the nurses’ responsiveness helped provide spiritual care, but they felt that they had an intuitive understanding of residents’ spiritual needs and how to address them.

### Spiritual care in terms of togetherness

The nurses strongly emphasized the importance of togetherness with residents as an important part of spiritual care. This idea is described in the subthemes ‘being present’ and ‘sensitivity in communication’.

### Being present

The nurses emphasized that their presence had some inherent qualities that were strongly connected to spiritual care. An active and healthy ‘presence’ was characterized by providing attitudes of empathy, love, reciprocity and sitting together in stillness or protecting the residents from intrusions when they needed quiet time. Such presence required much of the nurse’s time and was difficult to achieve. One way to achieve a sense of presence was to be consciously attentive to the residents’ requests for contact. A nurse described the intent of this presence:

B4: ‘I think it is important that we show that we are present and that they get the feeling that we want to help them if necessary.’ (p. 9)

Spiritual care was also characterized by conflict-free interactions between the nurses and the residents, which in turn created a tranquil atmosphere. The nurses reported that often, the residents were irritable in unpredictable situations. The nurses discussed the boundaries between appropriate closeness and distance because some residents did not want closeness in the form of a hug and often pulled away. The nurses believed that it was important to respect this choice. However, the nurses also wanted to include each resident in the community, which required knowledge about how the resident was functioning. One nurse reflected on the residents’ vulnerability, saying the following:

D5: ‘We are probably the most important people in their lives. It’s about being present where the residents are in life.’ (p. 15)

### Sensitivity in communication

The nurses considered spiritual care to be incorporated into different forms of communication. Good communication was described involving both the nurse and the resident as participants. The characteristics of good communication included the creation of a happy mood through small talk and being emotionally touched by the residents’ behaviour and emotive expressions. Because many residents showed strong emotions, such as joy, sorrow and despair, but were unable to describe their feelings, the nurses emphasized the need to accommodate the resident’s communication deficiencies in a timely manner by understanding the resident’s wordless communication. One nurse reflected on the meaning of her own behaviour when communicating with residents in the following quote:

C5: ‘You need to have peace in your body and see the resident so that you actually are listening to what they say and how they say it.’ (p. 14)

The nurses emphasized the importance of knowledge and experience in dementia care when caring for residents with language deficits. If the nurses used too many words in their explanations, the residents would respond with uncertainty and confusion because they did not understand what was said. In such situations, the nurses used hand gestures or body language to customize their communication with each resident.

### Spiritual care as providing meaningful activities for everyday life

Spiritual care created contentment in everyday life situations through meaningful events. The nurses used the resident’s surroundings to try to stimulate the residents’ own initiative while preserving the resident’s autonomy and ability to work with improved coping skills. This form of care was reflected in the following subthemes: ‘facilitating activities’ and ‘meeting the residents’ religious needs’.

### Facilitating activities

Spiritual care was associated with the facilitation of daily activities. The nurses sought to make life as meaningful as possible for the residents through recognition and participation. They attempted to adapt each resident’s surroundings to help individuals cope with activities that they had forgotten how to perform, including eating and dressing. One nurse said:

A2: ‘It is clear that although they are here and are demented, all is not lost. It’s very, very much left.’ (p. 9)

The creation of cultural activities was associated with spiritual care because the nurses believed that entertainment provided contentment in life that residents did not naturally have as a result of their dementia. Gentle music was beneficial for the residents because it relaxed them and sometimes even evoked language skills. The nurses invited the residents to take small excursions outside the main living area, such as walking in the garden, making Christmas cookies or waffles, weeding flower beds or reading newspapers together. Small adventures, such as going out into nature and hearing birds sing, listening to music, eating a good chocolate or having a religious experience enriched the residents’ daily lives. All of these activities involved sharing daily life at a level that residents could manage.

### Meeting the residents’ religious needs

The nurses’ understanding of spiritual care also encompassed activities involving the residents’ religious beliefs. Not much attention was given to religious care in the nursing home, and very few nurses openly stated that they were willing to pray with the resident. However, one nurse said that when she read the Bible to a worried resident, the resident immediately became calm. Another nurse emphasized the importance of preserving aspects of the residents’ previous lives that remained intact, saying the following:

J1: ‘Residents do not take the initiative themselves to meet their religious needs, but if it is offered as an invitation or a concrete clue, then they take it and enjoy much of it.’ (p. 17)

The nurses observed that if the resident had symbols in his or her room, such as a personal Bible or other scriptures or crosses or crucifixes on the wall, the nurses assumed that those symbols meant something to the resident. Furthermore, if residents wished to attend devotions or church and/or visit the cemetery, they received assistance.

## Discussion

The aim of this study was to investigate how nurses and care workers provide spiritual care for people with dementia in nursing homes. The findings of this study show that the nurses were unsure what spiritual care entailed. Through the focus group discussion, the nurses developed an understanding that spiritual care was integrated into general care in the form of physical touch, presence, the facilitation of meaningful activities and sensitivity in communication. The nurses also expressed that they had little theoretical knowledge about spiritual care, which resulted in a particular reluctance to care for residents’ religious needs. The nurses’ provision of spiritual care was based on intuition developed through experience with residents with dementia.

One question that emerged from the findings is “What do nurses mean when they perceive that spiritual care is integrated into general care?” The meaning of integrative spiritual care is that spiritual care pervades all dimensions of general nursing care, in line with the descriptions of holistic nursing care [8, 13]. This perspective seems to fit with the understanding of spiritual care described by the nurses in this study. Based on Sawatzky and Pesut’s [13] definition (presented in the introduction), integrative expressions of spiritual care rest on nurses’ awareness of the transcendent dimension. Transcendent awareness reflects an expectation that spirituality empowers and provides freedom and meaningful experiences in human lives [13]. According to Schnell [20], the transcendent dimension encompasses a vertical and a horizontal orientation. The horizontal orientation refers to a person’s expression of spirituality towards other people and/or their surroundings. The vertical orientation refers to a person’s expression of spirituality towards God/a deity [18, 20]. It seems relevant to state that the nurses were aware of the residents’ experiences of self-transcendence when they provided spiritual care. For example, the interpersonal relationship between residents with dementia and the nurses in our study was characterized by sensitivity in verbal and non-verbal communication, as the nurses emphasized the importance of observing residents’ body language. Albers et al. [46] underline the importance of non-verbal communication in the care of residents with dementia because many residents lose their language skills as the dementia progresses; sensitivity in communication is also important in response to residents’ cognitive impairments. The nurses in this study viewed spiritual care as emerging from the interaction between themselves and the person with dementia. The nurses characterized these relationships as being attentive, in the form of just being with the residents. Some studies emphasize connections through relationships as the very core of spirituality [20, 25, 47]. The importance of promoting a connection in the relationships between nurses and residents seems to be vital when providing for residents’ spiritual needs and has been confirmed in several studies [48–51].

The nurses in the current study who emphasized the creation of good relationships with residents may be seen as being altruistic in the sense of having a strong commitment and a desire to create wellness and meaningfulness for their residents. The nurses’ perspectives on spiritual care correspond with those of Carson and Stoll [52], who claim that residents’ experience of well-being is strongly associated with spiritual care; these perspectives also correspond with Sawatzky and Pesut’s [13] definition. However, it is interesting that spiritual care in the form of awareness of residents’ experience of transcendence was understood to only a limited extent. Furthermore, the nurses in this study struggled to address residents’ religious needs. This might have something to do with the nurses feeling that the Norwegian culture is not open to religious expressions in general and that this contributed to residents’ religious needs in the nursing homes not being adequately met. The nurses seldom discussed how residents’ religious beliefs influenced their meaningful experiences. The nurses felt that it was controversial to pray with residents but reported that some residents needed prayer to reach a state of peace. The nurses were afraid of making mistakes because of limited familiarity with a resident's religious life. Accordingly, they felt that the residents’ religious needs were difficult to meet. Spiritual care in dementia care means returning to the core values in the resident's life. Therefore, it is crucial that nurses collect information about the resident's life- history. A recognition of activities and people who have been important in their lives may help provide a sense of coherence and continuity for the residents [26, 27], and information from the resident’s family members, faith leaders or other people can offer vital information about the residents’ former interests. The nurses in this study said that they were aware of this, even if they only used this type of information to a limited extent.

Another interesting finding in our study is that the nurses’ spiritual care was based on intuition developed through experience working with residents with dementia. Intuition is described by Sawatzky and Pesut [13] as knowledge beyond what is considered logical and evident. Benner et al. [53] claim that expert care is characterized by intuition and the capability to safeguard the existential aspects of residents’ lives. Intuition emerges through theoretical and empirical knowledge integrated into nurses’ professional practice [53]. Nevertheless, the nurses in the current study admitted that they lacked experience and theoretical and practical knowledge about spiritual care, a response that is in line with the findings in other studies [54–57]. Furthermore, nurses found that they lacked a common language for this type of care and therefore met the residents’ spiritual needs using an intuitive approach.

It seems as if the nurses described intuition as a form of caring for the vulnerability and humanity in residents’ lives, much in line with Sawatzky and Pesut’s [13] understanding.

The theoretical approach and joint reflections among peers led to a greater understanding of the conceptual content of spiritual care among the nurses. The nurses felt that they were experts in general dementia care but were uncertain about their ability to provide spiritual and religious care. The findings of this study indicated that nurses need to reflect together about spiritual care to increase their awareness and knowledge of the topic, establish concepts and elicit a rationale for their shared practices.

### Limitations

The findings of this study cannot be generalized. Nevertheless, the provision of spiritual care by the nurses (RNs and care workers) might be recognizable in other international contexts because dementia itself is not context bound. Spiritual care is primarily about nurses encountering a fellow human being and is secondarily influenced by social assumptions and cultural barriers. In this way, the nurses and care workers’ descriptions of spiritual care may be familiar in other parts of the world. A limitation of this study is that few participants from countries other than Norway were in the sample. An additional limitation of the study relates to the high average age of the nurses and the very small number of men in the focus group interviews. These demographics are common in Norwegian nursing homes. The limited age distance between the nurses and the residents might have improved the nurses’ preconditioning to understand their residents’ cultural background and history. The imbalance between the sexes may have affected the results because of an over-representation of women. However, this is uncertain, and the findings might be close to the reality of nursing homes in a Scandinavian context.

## Conclusions

The nurses in this study considered spiritual care to be an integral part of general care for people with dementia. Furthermore, the nurses provided spiritual care intuitively, by providing togetherness and including their residents in meaningful activities. The dialogue in the focus group interviews revealed that the nurses increased their understanding of spiritual care during the focus group discussions. The joint reflection helped the participants find the words to describe spiritual care in their daily practice.

This study has noted that the implications for practice must be to facilitate arenas in which nurses can regularly reflect upon and discuss their professional understanding of spiritual care. Further studies on spiritual care are required to develop the necessary knowledge and skills for nurses in practice.

## Abbreviations

RN: registered nurse
LPN: licensed practical nurse
LSØ: Liv Skomakerstuen Ødbehr (first author)
KK: Kari Kvigne (second author)
SK: Solveig Hauge (third author)
LJD: Lars Johan Danbolt (fourth author)
